# High-Gain Wideband Circularly Polarised Fabry–Perot Resonator Array Antenna Using a Single-Layered Pixelated PRS for Millimetre-Wave Applications †

**DOI:** 10.3390/mi13101658

**Published:** 2022-10-01

**Authors:** Noureddine Melouki, Abdesselam Hocini, Fatima Zahra Fegriche, Peyman PourMohammadi, Hassan Naseri, Amjad Iqbal, Tayeb A. Denidni

**Affiliations:** 1 Centre-Energie Matériaux et Télécommunications, Institut National de la Recherche Scientifique, Montreal, QC H5A1K6, Canada; 2 Laboratoire d’Analyse des Signaux et Systèmes, University of M’Sila, M’Sila 28000, Algeria

**Keywords:** Mm-wave, high-gain, wideband, Fabry–Perot Resonator Antenna (FPRA), partially reflective surface (PRS), array

## Abstract

In this paper, a wideband and high-gain circular polarised Fabry–Perot Resonator Antenna (FPRA) with a single partially reflective surface (PRS) layer is automatically generated and optimised using a VBA-based interface system between CST Microwave studio and Matlab. The proposed PRS layer is a promising superstrate for wideband and high-gain FP resonator antennas due to its relatively high reflection coefficient magnitude and positive phase gradient, which resemble that of the optimum PRS over the relevant frequency band. The circular polarisation was achieved using a sequential feeding network for a 2 × 2 array air-gapped slot-coupled elliptical patch antenna. The proposed design achieved an impedance bandwidth of 48.58% (15.3 GHz) ranging from 23.84 GHz to 39.14 GHz, and the −3 dB gain bandwidth was 22.42% (6.25 GHz) from 24.75 to 31 GHz, with a peak gain of 17.12 dB at 29 GHz, and an axial ratio bandwidth of 21.75% (6.2 GHz). In addition, the achieved radiation efficiency was 90%. Consistent and almost invariant radiation patterns are achieved over the millimetre-wave frequency band of interest. The experimental and simulated results are in good agreement, justifying the feasibility of the proposed design as a high-gain and wideband FP resonator array antenna for Mm-wave applications.

## 1. Introduction

Wideband and highly directive antennas are becoming popular topics in both the academic and industrial sectors, particularly in the upper microwave region and the untapped millimetre wave spectrum, due to the growing demand for high-data rates and low-latency wireless communication systems [1]. Traditional technologies have been used to design these types of antennas, including reflectors, waveguide horn antennas, and microstrip-fed patch arrays [2,3,4]. However, the aforementioned technologies have significant drawbacks, including design complexity, high fabrication costs, and feeding-network-induced losses.

To address the aforementioned drawbacks, one approach is to use a Fabry–Perot Resonator Antenna (FPRA) [5], which consists of a Partially Reflective Surface (PRS)-based superstrate placed at a distance (usually half a wavelength) from the ground plane, creating an air-gapped cavity, and excited by a feeding source antenna (single or array) backed by the ground-plane. This type of technique is a simple and cost-effective way to achieve significant improvements in terms of antenna radiation characteristics [6,7,8,9]. However, this type of antenna suffers even more from the inherent narrow bandwidth because the PRS layer properties, which are what primarily determine the antenna’s characteristics, such as its frequency, radiation patterns, gain, and bandwidth, invoke the typical narrow band cavity [10].

To solve this problem, numerous research projects have been carried out to increase the operation bandwidth and peak gain performance by using Frequency Selective Surfaces (FSS). As in the case of [11], where a dual-band fractal FSS for the 5G lower band frequencies of 750 MHz and 3.5 GHz, has been proposed as a base model to be tweaked to any operating frequencies by simply solving a 2 × 2 linear equations system, which make it a versatile FSS design that can be used as a reflector, such as in the case of [12], where an FSS-based reflector is used to enhance both the bandwidth and gain of a 1 × 4 rectangular microstrip array antenna, with a fractional bandwidth of 51.12% corresponding to 2.3 GHz in the frequency range of 3.5 to 5.8 GHz. In addition, the maximum achieved peak gain was 12.4 dBi, with a 4.4 dBi improvement in comparison to the antenna without the FSS.

In [13,14], multi-layer superstrates based on periodically printed FSS arrays were used to improve bandwidth on single and dual-band applications, whereas in [15], a dielectric-based one was used to achieve a broadband EBG resonator. In addition to increasing bandwidth, using multi-layer superstrates increases the antenna’s profile and makes it a bulky structure. Many researchers have been interested in a more practical method that uses double-sided printed metallic layers on a dielectric medium [16,17,18]. This superstrate generates a positive reflection phase gradient over a specific spectrum of frequencies to widen the −3 dB gain bandwidth of the FP resonator antenna while maintaining a degree of compactness and low profile [19] as the height of the resonance cavity is only a half wavelength. This design offers both high gain and a low fabrication cost.

Additionally, circular polarised (CP) antenna arrays are in high demand due to their decreased polarisation mismatch and immunity to Faraday rotation [20,21]. This principle also holds true for both Mm-wave communication systems and Indoor Wireless Communication (IWC) technology, particularly in crowded indoor conditions, where CP enhances channel performance by reducing multi-path interference, low absorption losses, and signal attenuation [22,23,24]. In [25], the authors present interesting research on waveguide indoor antennas as well as the two most common approaches to planar indoor antennas, namely broadband CP printed monopole antennas (BCPPMAs) and broadband CP printed slot antennas (BCPPSAs), as Broadband Circular Polarised Printed Antennas for Indoor Wireless Communication Systems.

A wide impedance and axial ratio have been achieved in [26] using an Archimedean spiral radiator with a ring-slot structure, but the broadside beam is wide and tilted. In [27,28], various works on CP antenna arrays for millimetre-wave radiation were presented high gain and good impedance matching and were made possible through SIW and multi-layer PCB technology for slot-array cavity-backed antennas. The practical applications, however, might be hindered by their complicated antenna structure configurations.

The primary source of gain loss and unwanted radiation in Mm-wave array antennas is the feeding network. Sequential feeding can be used for sub-arrays to increase bandwidth and decrease antenna gain losses. When ideal array elements are used with the correct amplitude and phase, it has been asserted that the axial ratio of a sequential feeding network is frequency-independent [29]. Numerous works [30,31] have examined the effectiveness of the sequential feeding network for improving CP, where [32] compared DRA arrays and patch antenna arrays using a variety of sequential feeding networks.

This study uses a VBA-based interface mechanism between CST Microwave Studio and Matlab to automatically design and tune a wideband and high-gain 2 × 2 sequentially fed circular polarised Fabry–Perot Resonator Antenna (FPRA) with a single partially reflective surface (PRS) layer. The paper is structured as shown below:

The suggested antenna operation principle is described in Section 2 and is based on the ray tracing analysis using a single PRS layer. In Section 3, the geometry, design process, and the results of the single Fabry–Perot antenna are covered. In contrast, Section 4 and Section 5 detail the 2 × 2 sub-array element CP antenna and its performance characteristics as well as a comparison of the suggested design to other designs. Finally, conclusions on the matter are summarised in Section 6.

## 2. Proposed Wideband Single-Layered PRS FPRA Antenna

The Fabry–Perot resonator antenna (FPRA) is regarded as a highly directive antenna [1]. It is formed of a simple radiating source, a ground plane, and a PRS. Gain and directivity enhancements are possible when the separation $h_{c}$ between the ground and the PRS fulfils the cavity resonance condition in accordance with ray-tracing analysis [32], in which the reflected waves from the ground and the PRS’s reflection phase response are in phase, resulting in the maximum gain in the broadside direction, and the spacing $h_{c}$ equals the following:

(1) $$h_{c}=\frac{c}{4\pi f_{r}}\left(\varphi_{PRS}+\varphi_{GND}-2N\pi \right)$$

where $\varphi_{PRS}$ and $\varphi_{GND}$ are both the reflection phases of the PRS and the ground plane, respectively. The number *N*, which equals 0, 1, 2, etc., designates the resonance mode order, and it is set to 0 for a low profile Fabry–Perot-based resonator antenna. $f_{r}$ and c are the resonance frequency and the speed of light in the vacuum, respectively.

The FPRA antenna’s directivity, assuming the source antenna’s directivity is $D_{ref}$ at a given frequency $f_{r}$, would be the sum of this latter and the PRS directivity $D_{PRS}$, which is written as:

(2) $$D_{PRS}=10\log\left(\frac{1+\Gamma }{1-\Gamma }\right)$$

where Γ denotes the PRS superstrate layer’s reflection magnitude, and the total theoretical FPRA directivity is formulated as follows [32]:

(3) $$D_{FPRA}=D_{ref}+D_{PRS}$$

It is evident from Equations (2) and (3) that the directivity of the FPRA is positively correlated with the PRS reflection amplitude and that the higher this latter value is, the better the overall directivity of the FP resonator antenna.

Conventional FPRAs have a limited radiation bandwidth because the cavity spacing *h_c_* and partially reflective surface phase $\varphi_{PRS}$ are frequency-sensitive.

The following formula, which assumes that the dielectric slab is lossless, can be used to approximate the FPRA’s aperture surface for the required directivity [33]:

(4) $$A=\frac{10^{\frac{D_{FPRA}}{10}}\lambda^{2}}{\pi^{2}}$$

where λ is the operating wavelength of the FPRA.

With a PEC ground plane ($\varphi_{GND}=\pi$), Equation (1) can be rearranged to represent the PRS reflection phase as follows:

(5) $$\varphi_{PRS}=\frac{4\pi h_{c}}{c}f_{r}+\left(2N-1\right)\pi$$

According to Equation (5), if the PRS reflection phase increases with the resonance frequency *f_r_*, the FPRA will operate in a wider frequency band with a higher gain bandwidth. To design a wideband and high-gain Fabry–Perot-based Resonator Antenna, a PRS with a positive reflection phase gradient is required.

### 2.1. Proposed PRS Structure

**Figure 1:**
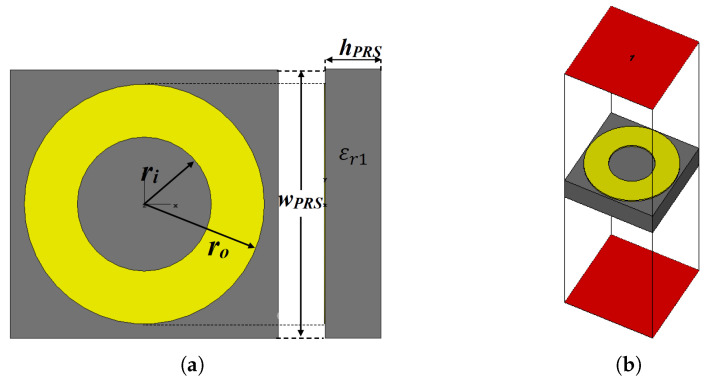
(**a**) Geometry of the initial PRS unit cell; (**b**) analysis setup of the unit cell (CST).

In the previous section, it was discussed that the main key point for designing a wideband FP resonator antenna is a PRS with a positive phase gradient, and to that end, a double-sided layer with Frequency Selective Surface (FSS) printed patterns is proposed and designed on a Rogers RT/duroid 5880 dielectric slab ($\varepsilon_{r1}$ = 2.2, tanδ=0.0009), with a thickness *h_PRS_* of 0.79 mm. The lower part is a simple ring-shaped FSS with the following design parameters: *w_PRS_ = 3.8* mm, *r_o_* = 1.7 mm, *r_i_* = 0.95 mm and *g* = 0.4 mm. The upper part is synthesised automatically using an automated system built by implementing an interface between CST Microwave studio and the embedded genetic algorithm in Matlab via a VBA-based link. Figure 1a shows the geometry of the initial PRS unit cell.

The setup for analysing reflection phase characteristics is shown in Figure 1b, where boundary conditions are applied as follows: along the ±x-axis, the Perfect Magnetic Boundary condition (PMC) is used, while in the case of the ±y-axis, the Perfect Electric Boundary condition (PEC) is applied. Two wave ports are set at a distance of λ/2, from the unit cell under investigation, along the z-axis, with an open boundary condition, so only the normal incidence is taken into account.

**Figure 2:**
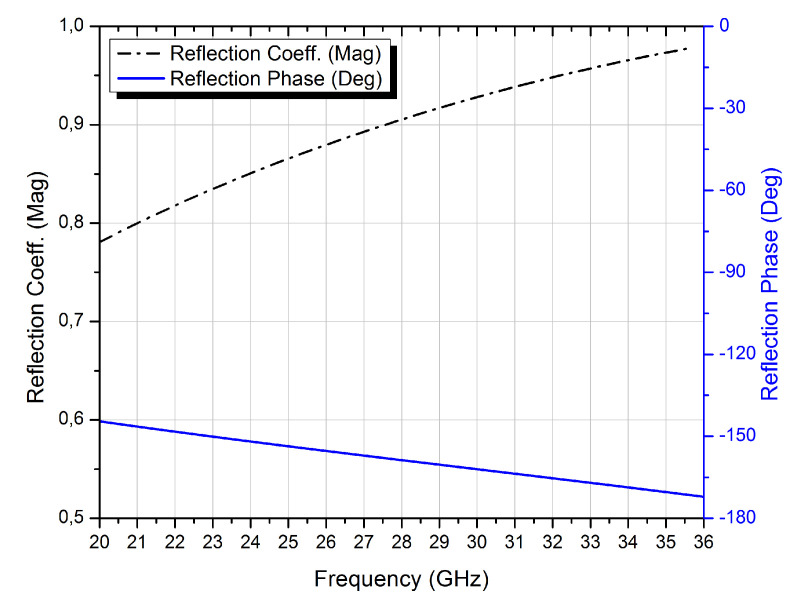
Reflection phase and magnitude of the initial PRS unit cell.

Figure 2 depicts the simulated magnitude and phase of the reflection coefficient response of the initial PRS unit cell. At 28 GHz, the PRS unit cell is highly reflective around the frequency band of interest, with a magnitude of Γ=0.9. A high-gain antenna is designed to have a high reflection magnitude. However, as shown in Figure 2, the reflection phase of the unit cell decreases as the frequency increases, resulting in a narrower bandwidth. To broaden the antenna bandwidth while maintaining high-gain performance, a new unit cell with a positive reflection phase gradient is proposed.

The following method is based on the same PRS unit cell and employs a pixelated-based pattern on the other side of the unit cell to generate a positive phase gradient that follows the optimal phase, thereby increasing bandwidth.

The upper side of the PRS structure is the targeted area for this procedure, which is created using the well-established VBA-based connection between CST Microwave studio and MATLAB’s embedded genetic algorithm (GA). Each PRS unit cell is divided into n × m pixels. These pixels are defined using binary encoding, where a value of 1 or 0 denotes the presence or absence of copper on the pixelated sub-cell. It is impractical to solve this problem by increasing the number of pixelated cells because doing so increases the total number of possible structures. Instead, a confined search space global optimisation solution is required to identify the best candidate using a predefined fitness function, and the genetic algorithm is an efficient method for doing this.

It was discovered that genetic algorithm (GA)-based optimisations have a lot of potential for developing novel solutions to EM-based problems. In [34], an EBG structure was optimised using a genetic algorithm to increase gain and bandwidth, whereas [35] used the same technique to optimise an AMC-based reflector to achieve a significant improvement in the peak gain and front-to-back ratio, along with a low side-lobe level (SLL), for 5G applications. On the other hand, a compact UWB FSS structure was developed in [36] using a similar GA-based synthesising system, with specific parameters in terms of the targeted structure and the fitness function, completely different from the current work objectives. This was conducted in order to increase the gain of a UWB monopole antenna.

The fitness function in the proposed designing scenario is defined as the root-mean-square error (RMSE) between the reflection coefficient phase response of the generated PRS unit cell under study and the optimum phase and is defined as follows:

(6) $$Fit=RMSE(Phase_{PRS},Phase_{Optimum})$$

where RMSE is defined as:

(7) $$RMSE=\sqrt{\frac{\sum_{n=1}^{N}\left(Phase_{PRS}-Phase_{Optimum}\right)}{N}}$$

The reflection coefficient phase of the PRS unit cell is taken at equal frequencies $f_{i}$, with the total number of N=1001 frequency points, within the designated overlapping frequency band of interest ($f_{min}$⩽$f_{i}$⩽$f_{max}$).

**Figure 3:**
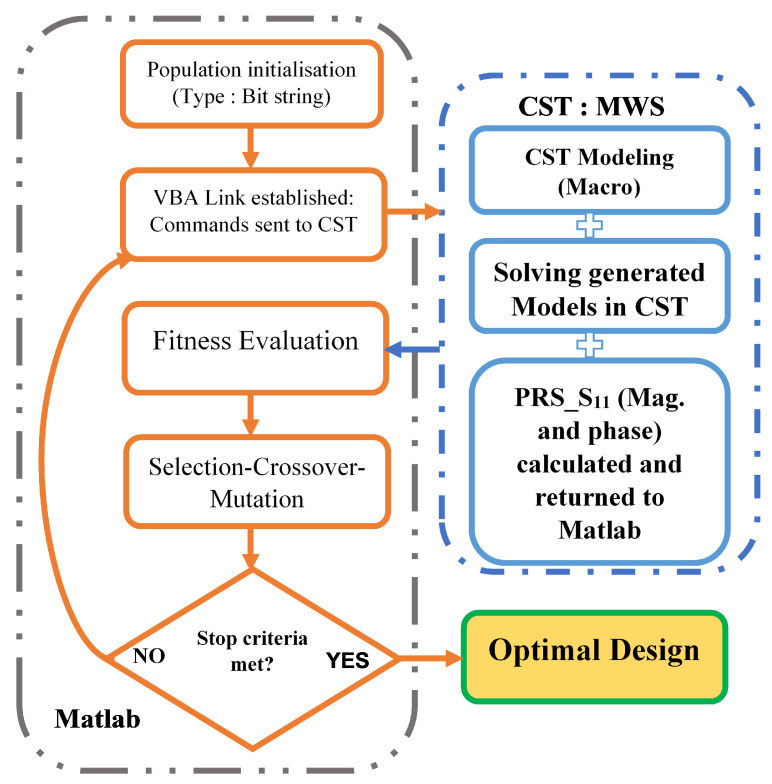
The Proposed flowchart of the GA optimisation process for the PRS unit cell.

Figure 3 depicts the flowchart of the GA-based optimisation process for a positive phase gradient PRS unit cell. As a starting point, the PRS unit cell is discretised into 12 × 12 pixels, yielding a resolution of 0.31 × 0.31 mm while accounting for fabrication constraints and maintaining geometrical flexibility.

**Figure 4:**
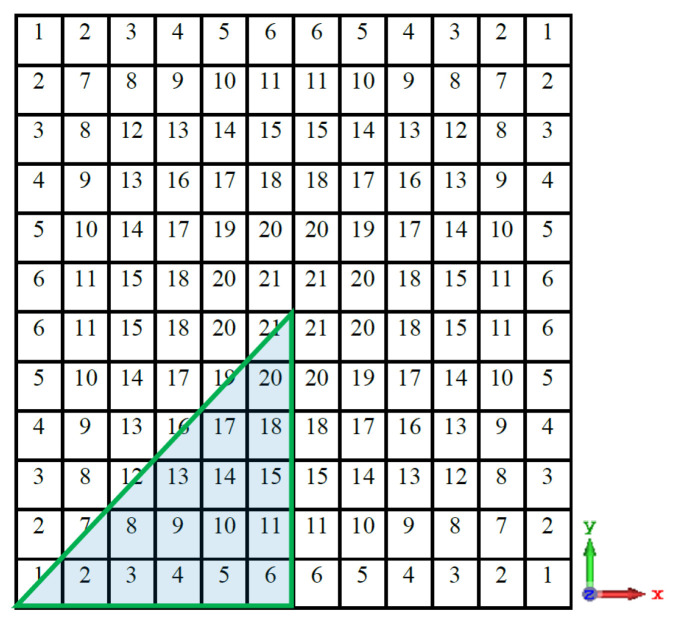
The proposed four-folded symmetry pixelated PRS unit cell.

The necessity for 144 binary encoded pixels to represent a 12 × 12 discretised unit cell created a fairly large search space, making it impractical and time-consuming to find the best solution. In order to get around this, a four-folded symmetry is imposed, as seen in Figure 4. As a result, the search space for the ideal solution (the number of bits needed to represent the PRS unit cell) is drastically reduced to 21.

By using the suggested genetic algorithm, with a uniform mutation rate of 0.001, a single point crossover, and the tournament selection as its parameters, the proposed optimisation process attempts to manipulate the reflection coefficient phase of the potential PRS design candidates in order to produce a PRS unit cell with a phase that closely resembles the positive gradient optimum phase within the frequency band of interest.

The number of iterations (generations) is set to 20, and the population size is set to 200 different binary strings, each of which represents a distinct pixelated PRS unit cell. A new population of 200 PRS candidates going through the same evaluation process is created after all 200 initial pixelated PRS structures have been evaluated and ranked, and the best ones have been chosen for the crossover and mutation processes. The latter is repeated as necessary to achieve the desired fitness value (*Fit* tends toward zero) or until the assigned number of repetitions has been reached.

**Figure 5:**
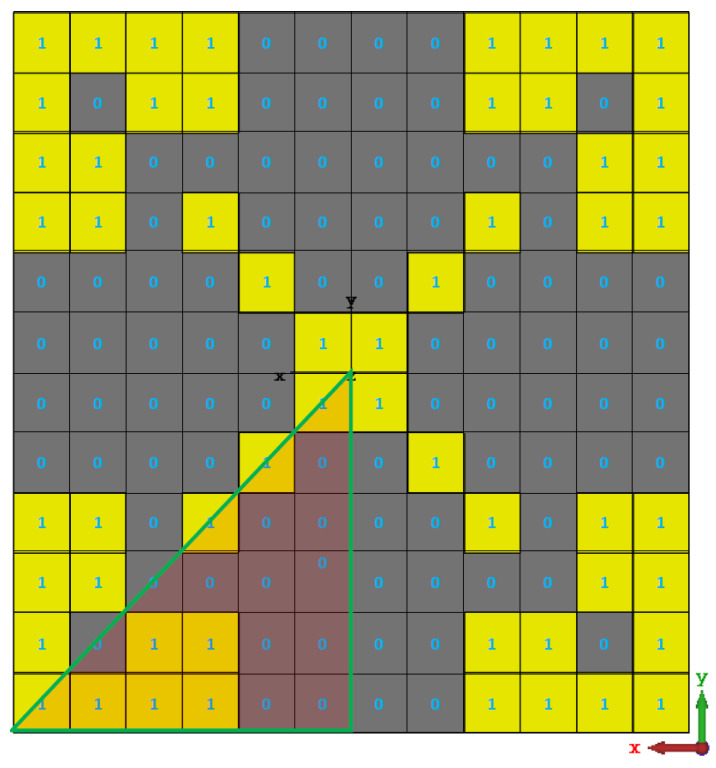
The optimal PRS design’s binary representation.

**Figure 6:**
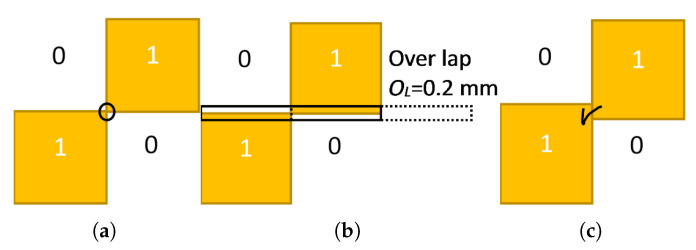
(**a**) Combination with infinitesimal connection; (**b**) proposed overlapping scheme; (**c**) a good connection with the overlapping area.

At the 12th iteration, when the stop criterion is satisfied, the best PRS design is obtained, as shown in Figure 5. However, this design has an infinitesimal connection issue between two sub-patches when these constellations $\left(\frac{10}{01}\right)$ or $\left(\frac{01}{10}\right)$ are present, which could result in a malfunctioning PRS structure because of fabrication tolerances (Figure 6) [36].

To get around this inconvenience, adjacent sub-patches are then overlapped to ensure electrical contact, but this must be carried out while considering how the overlapping area will affect the overall structure. As a result, a minimum value for the overlapping area is required, and this value is naturally constrained by the machine precision used in the fabrication process. The overlapping offset is configured to $O_{l}=0.2$ mm.

**Figure 7:**
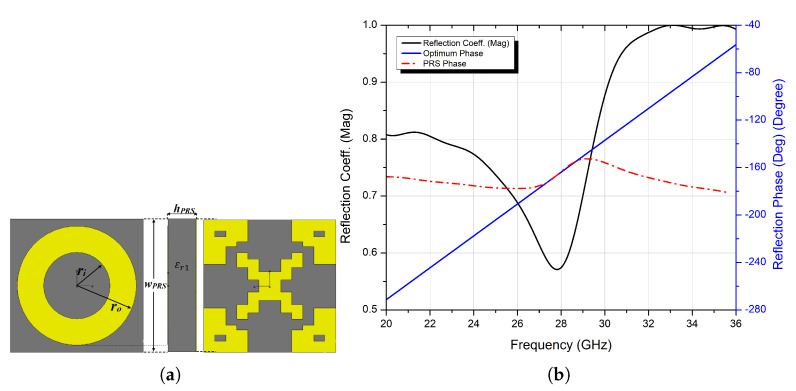
(**a**) The final optimised PRS design, and (**b**) reflection coefficient’s magnitude and phase of the proposed PRS unit cell.

Figure 7a shows the final GA optimised and ready for fabrication design. The PRS unit cell also exhibits polarisation insensitivity, making it applicable to circularly polarised antennas because the design enforces the four-folded symmetry.

The binary string of the final optimised PRS unit cell is:

$$B^{PRS}=\left\{1\;1\;1\;1\;0\;0\;0\;1\;1\;0\;0\;0\;0\;0\;0\;1\;0\;0\;1\;0\;1\right\}.$$

The resulting reflection coefficient phase of the optimised PRS has a positive gradient and almost perfectly resembles that of the optimum PRS over a wide bandwidth spectrum range, with a relatively high reflection magnitude (see Figure 7b), and this can be a crucial key point in designing a wideband with high-gain FPRA antenna since higher reflective PRS leads to higher gain as per Equations (2) and (3) but narrower −3 dB bandwidth, and to broaden this, a PRS with a positive phase gradient is a must to achieve that goal, as per Equation (5). The proposed PRS design can be a potential superstrate candidate for a wideband and high-gain FP resonator antenna, and this can be seen in the following sections.

### 2.2. Feeding Antenna

A well-designed feeding antenna is a crucial component of designing a high-gain and wideband FPRA antenna because the feeding source antenna for an FPRA is as important to its performance as the PRS structure. Due to its low profile, simplicity in fabrication and feeding, stable broadside radiation, and capacity for wide bandwidth, an air-gapped slot-coupled patch antenna makes a good candidate for a high-gain and wideband FPRA antenna.

**Figure 8:**
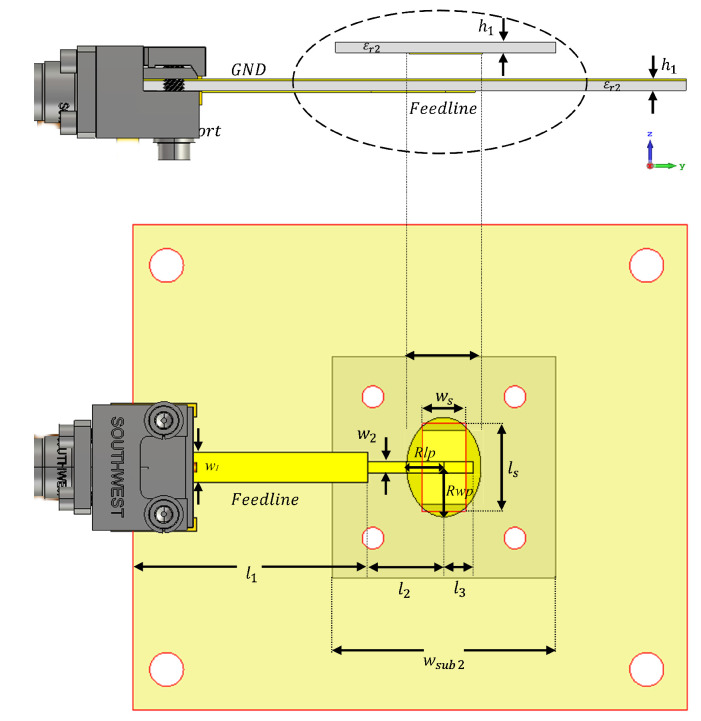
The schematic diagram of the slot-coupled elliptical patch feeding antenna.

Figure 8 illustrates the suggested feed antenna design. It is made up of a parasitic patch that is coupled to a feeding line via a slotted ground plane (bottom layer) and separated by an air-gap *h_air_* of 1 mm for the suppression of surface waves, as the latter can affect the FRPA cavity’s performance [37]. The bandwidth is increased by adding an impedance matching network to the feedline. The substrate for the parasitic patch and the bottom layer (ground plane and feed line) is made of the Rogers RO3003 material, which has the following specifications: thickness (*h_s_*) = 0.254 mm, permittivity $\varepsilon_{r2}$ = 3.0, and loss tangent of 0.001). Table 1 lists a summary of the antenna design parameters.

**Figure 9:**
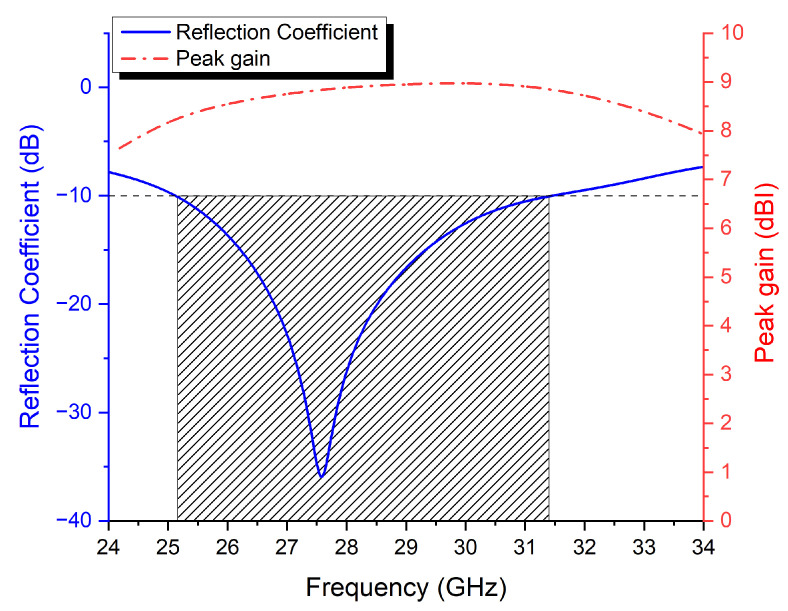
The simulated results of the slot-coupled patch antenna reflection coefficient and peak gain.

Figure 9 shows the simulated reflection coefficient (S_11_) and maximum gain of the feed antenna. It can be seen that the impedance bandwidth covers the Mm-band spectrum, ranging from 25.1 GHz and 31.49 GHz. The peak gain was 8.9 dBi within this band, making it a good candidate to be used as a feeding source for the proposed FRPA antenna, which will be designed in the following section.

## 3. Design and Characterisation of the Single-Element Antenna

**Figure 10:**
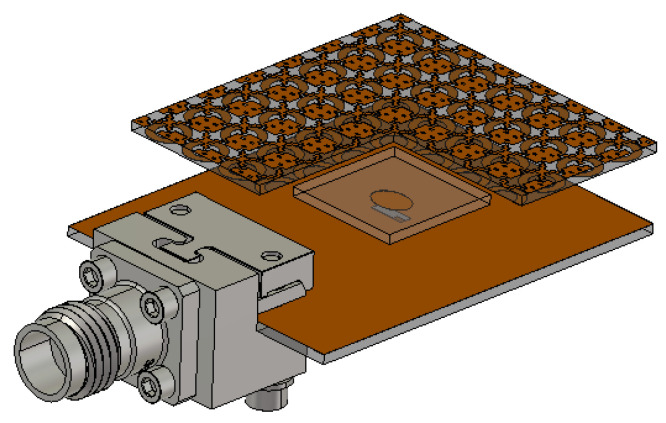
The proposed wideband FPRA design.

In this section, the optimised PRS structure (Figure 7) is applied as a superstrate layer to the proposed feeding antenna from the previous section (Figure 8), resulting in the proposed wideband and high-gain FP resonator antenna depicted in Figure 10.

The PRS superstrate is suspended over the shared ground plane at a distance *h_c_* using M2 nylon screws at the four corners, which are also considered during the simulation process, along with a 2.92 mm (K) Connector modeled in CST Microwave studio. A preliminary estimation of *h_c_* can be obtained by combining the simulated results of the suggested PRS unit cell reflection coefficient phase ($\varphi_{PRS})$ in Figure 7b and Equation (1), where $f_{r}$ is the FPRA’s centre frequency and $\varphi_{PRS}$ is also taken at that frequency.

The PRS reflection coefficient phase at the centre frequency of 28 GHz is approximately −163.5° (Figure 7b), and using Equation (1), the spacing *h_c_* is approximately 5.6 mm. Further optimisation is required to determine the appropriate distance for the Fabry–Perot Resonator Antenna’s best performance.

The wavelength λ is taken at the antenna’s lower end frequency (25.1 GHz), which is 11.95 mm, and the corresponding estimated FPRA directivity (*D_FPRA_*) is approximately 16.53 dBi using both Equations (2) and (3). As a result, the aperture size of the proposed FPRA antenna is estimated using Equation (4), and it is approximately 25.52 mm × 25.52 mm. In order to accommodate the array of 7 × 7 PRS unit cells that make up the proposed FP resonator antenna, whose effective size is 3.8 mm, a PRS structure with an aperture size of 26.6 mm × 26.6 mm is used as the superstrate.

**Figure 11:**
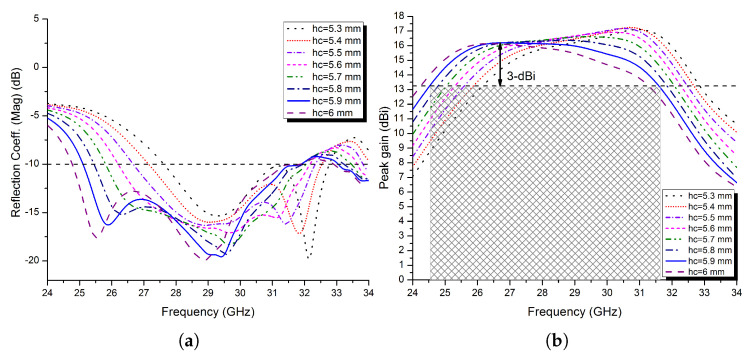
(**a**) The simulated reflection coefficient of the proposed FPRA and (**b**) peak gain at different spacing *h_c_*.

Figure 11a shows the calculated reflection coefficient S_11_ of the suggested FPRA using the previously estimated parameters, namely the aperture size (26.6 mm × 26.6 mm) and the cavity air gap *h_c_* = 5.6 mm. The impedance bandwidth (|S11|<−10 dB) of the proposed FPRA is approximately 5.97 GHz, spanning the range of 26.2 to 32.17 GHz. The maximum achieved peak gain was 16.97 dBi at 31 GHz.

In order to design a wideband antenna with high gain, the air-gapped cavity of the FP resonator antenna is a crucial component. As a result, the impact of altering this parameter on the proposed antenna’s overall performance is investigated.

To see the impact of the cavity height variation on the S_11_ impedance bandwidth, the peak gain, and the −3 dB gain bandwidth of the FPRA, all other parameters are kept constant while the cavity height is varied in steps of 0.1 mm from 5.3 to 6 mm. The results are shown in Figure 11a,b, and they are summarised in Table 2. From there, it is observed that, as would be expected from an FP resonator antenna, as the cavity thickness increases, the impedance and −3 dB gain bandwidths progressively increase while the peak gain gradually decreases.

Based on the results shown in Figure 11a,b, as well as Table 2, it can be said that the optimal cavity height for a high-gain FP resonator antenna with a wider operating bandwidth is 5.9 mm. At this height, the proposed FPRA demonstrated exceptional impedance bandwidth performance of 23.86% between 25.1 and 31.9 GHz and −3 dB gain bandwidth of 25.42% (24.5 to 31.63 GHz), with a maximum peak gain of 16.21 dBi.

**Figure 12:**
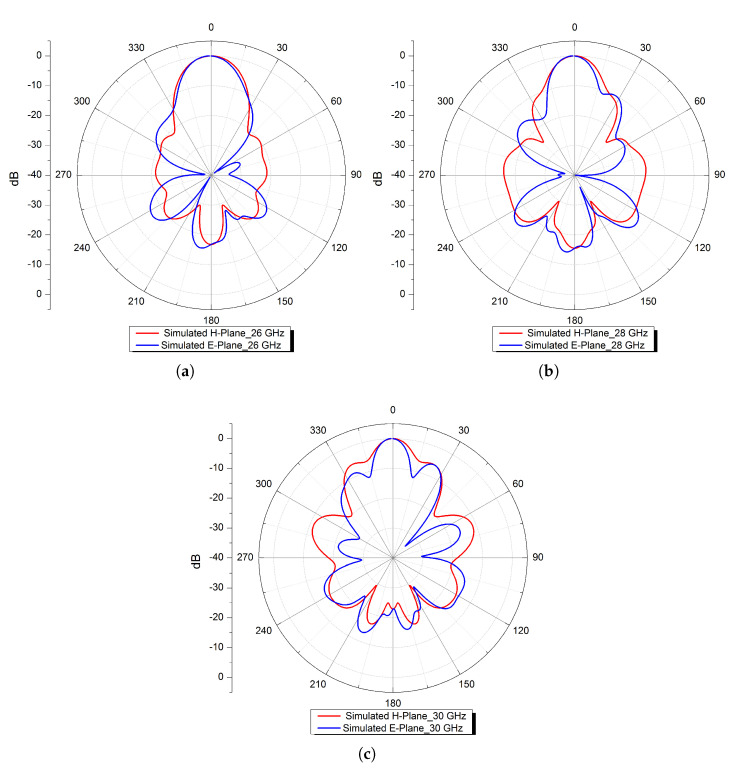
Normalised simulated radiation patterns in the E− and H−planes at (**a**) 26, (**b**) 28, and (**c**) 30 GHz.

Furthermore, radiation patterns at three distinct frequencies (26, 28, and 30 GHz) in both the E− and H−planes are simulated and shown in Figure 12. The patterns are of a directional nature with narrower beam-widths.

## 4. Design and Characterisation of 2 × 2 Elliptical Patch Antenna Sub-Array

Wide circular polarisation (CP) operation is targeted and accomplished using a sequential feeding network. In a sequential-like rotation structure, the examined 2 × 2 elements are fed one after the other with a 90° phase difference between them. The next subsections take into account and study the right-hand circularly polarised (RHCP) feeding network.

### 4.1. Sequential Feeding Technique for 2 × 2 Sub-Array

**Figure 13:**
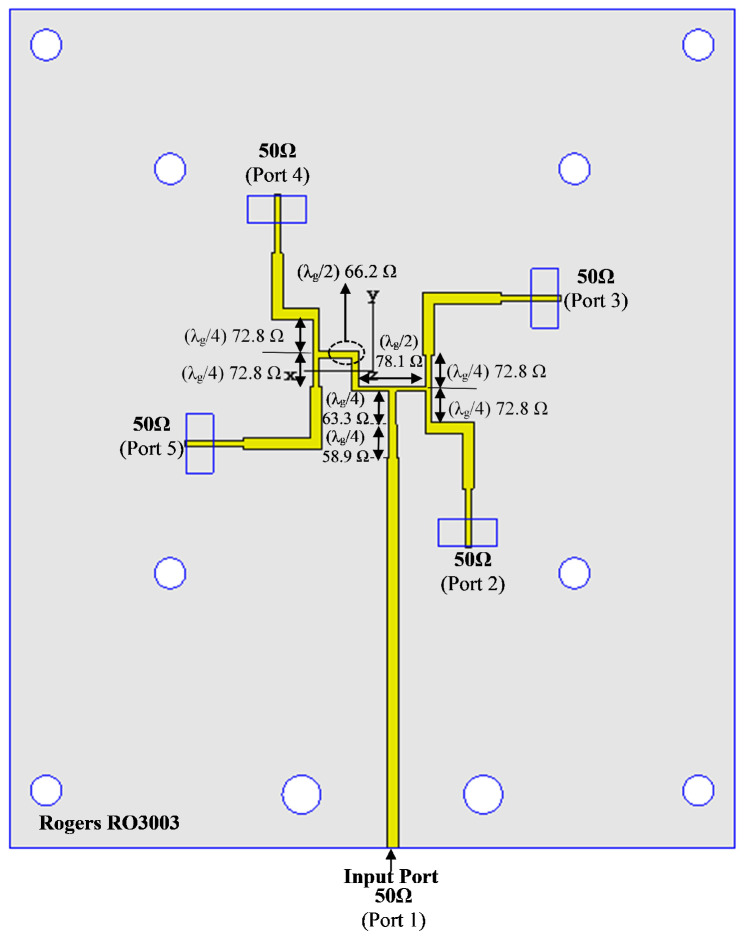
The proposed sequential parallel feeding network (RHCP).

As a sequential feeding network for RHCP operation, a parallel feeding mechanism is utilised in this section and is shown in Figure 13. The parallel feeding network is composed of three parallel T-junction elements, one of which has an anti-phase feeding network and the other two of which have 90° phase differences in feeding networks [31].

An anti-phase equal power divider, which creates a 180° phase difference between the output ports, forms the initial component of the feeding network. A power divider is connected to each of the anti-phase divider’s output ports with a 90° phase shift between the two output ports. Four output ports with a 90° phase delay are arranged counterclockwise in this feeding network. The single-element elliptical slot-coupled patch element was first rotated by 45° in an attempt to achieve CP operation, and these 2 × 2 elements were then rotated at 90° and are excited with a 90° phase shift in the counterclockwise direction. All design parameters of the proposed sequential feeding are shown in Figure 13 and were summarised in Table 3.

**Figure 14:**
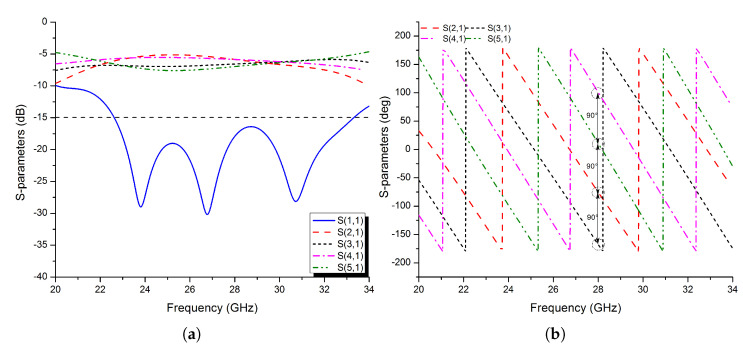
The simulated S−parameters of the proposed feeding network: (**a**) amplitude; (**b**) phase.

Figure 14a,b show the simulated amplitude and phase of the S-parameters of the parallel feeding structure. The parallel feeding network’s reflection coefficient (|S11|<−15 dB) ranges from 25 to 35 GHz. Over a wide frequency range, the maximum imbalance amplitude difference provided between ports 2–5 is less than 1 dB. Figure 14b clearly shows that each four-port has a nearly 90° phase difference with each other over the desired band.

### 4.2. 2 × 2 Antenna Array Configuration

**Figure 15:**
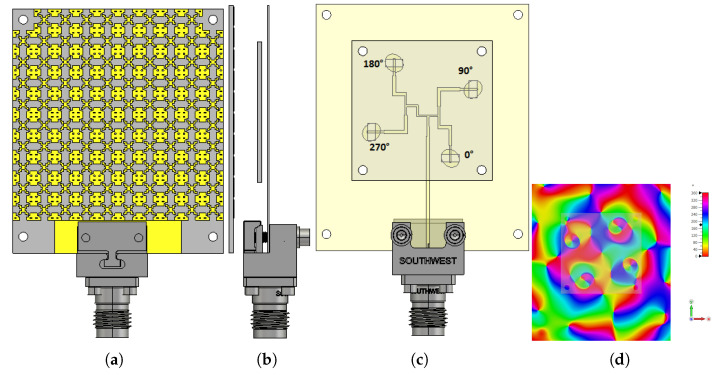
The proposed RHCP wideband FPRA design,(**a**) front view, (**b**) side view, and (**c**) back view; (**d**) its e-field distribution at 28 GHz.

In this section, the final optimised wideband and high-gain FP resonator antenna depicted in Figure 10 is used here as the base model for the 2 × 2 Sub-array CP antenna, where the elliptical patch is rotated first by 45° and fed by the same rectangular etched slot in a sequential-like rotation structure using the feeding technique for RHCP from the previous section (see Figure 13), so the 2 × 2 elements are fed one by one with a 90° phase shift between them. Moreover, a 10 × 10 PRS unit cell array was chosen for the final design, yielding a total size of only 38 mm × 38 mm for a 2 × 2 sub-array structure (see Figure 15)

As per previous sections, the spacing *h_c_* is a key point parameter in designing a high and wideband FPRA antenna. The same thing is expected with using the sequential feeding technique for CP operation. At first, *h_c_* is taken as half the wavelength λ/2 at 28 GHz, which is approximately equal to 5.35 mm and changed to 6 mm in steps of 0.1 mm, while keeping the other parameters constant, to determine the appropriate distance for the Fabry–Perot Resonator Antenna’s best performance in terms of impedance bandwidth, axial ratio (AR), and −3 dB gain bandwidths.

**Figure 16:**
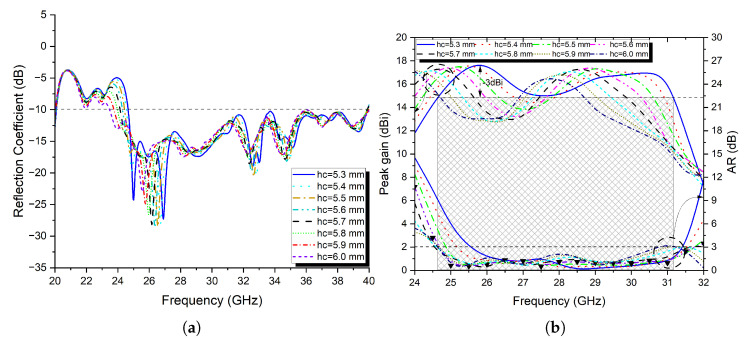
(**a**) The simulated reflection coefficient of the proposed FPRA and (**b**) peak gain and axial ratio at different spacing *h_c_*.

The simulated results are shown in Figure 16a,b. From there, it is observed that, as the cavity thickness increases, the impedance and AR bandwidth progressively increase, while the −3 dB gain bandwidth gradually decreases.

Based on the results shown in Figure 16a,b, it can be said that the optimal cavity height for a high-gain FP resonator antenna with a wider operating impedance and AR bandwidths is 5.6 mm. At this height, the proposed FPRA demonstrated exceptional impedance bandwidth performance of 48.53% between 24.3 and 39.87 GHz and an AR bandwidth of 25.13% (24.67 to 31.76 GHz), with a maximum peak gain of 17.66 dBi.

**Figure 17:**
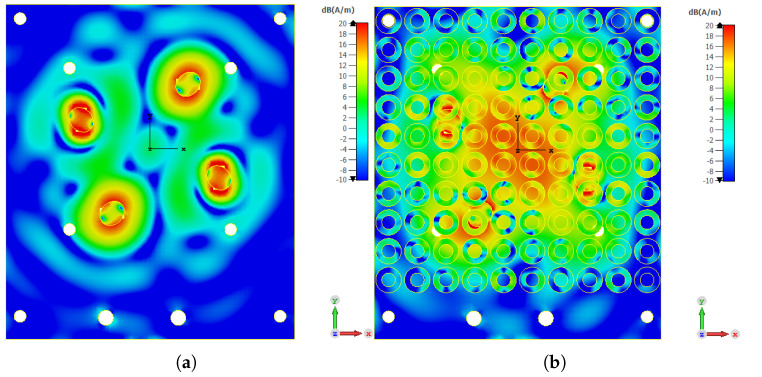
The simulated surface current distribution of the proposed FPRA (**a**) without and (**b**) with PRS superstrate at 28 GHz.

At 5.3 mm, the proposed antenna achieved its best −3 dB gain bandwidth of 23.65% between 24.6 and 31.2 GHz. The impedance bandwidth was 47.25% (24.65 to 39.9 GHz), and the AR bandwidth ranged from 25.5 to 31.52 GHz (21.12%), with a maximum peak gain of 17.72 dBi. So a trade-off between the −3 dB gain and axial ratio bandwidths. To that extent, a spacing *h_c_* of 5.3 mm is chosen here to achieve both capabilities. Moreover, by using the proposed PRS layer, the surface current distribution is saturated and focused in the broadside of the antenna in comparison to the design without PRS, as seen in Figure 17a,b.

Furthermore, radiation patterns at three distinct frequencies (26, 28, and 30 GHz) in both the E− and H−planes are simulated and shown in Figure 20, where it can be noted that these patterns are of a directional nature with narrower beam-widths.

## 5. Fabrication and Measurement Results of the 2 × 2 Elliptical Patch Antenna Sub-Array

**Figure 18:**
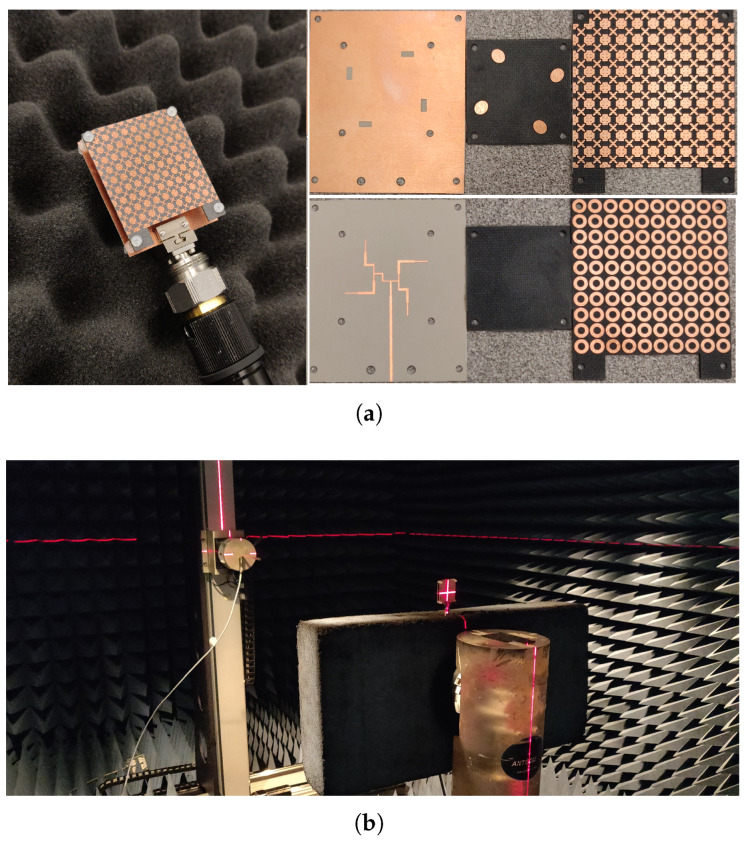
(**a**) The final fabricated antenna and its assembly parts and (**b**) far-field measurement setup.

The prototype validation process is carried out here by first fabricating all involved parts on their corresponding substrates and assembling them using M2 nylon metric pan head screws with the proper hex nuts to form the final proposed prototype (see Figure 18a).

As per the previous section, the optimal cavity spacing was fixed to 5.3 mm for further validation and analysis of the results. It is fed by a 2.92 mm (K) Connector on the feed line with an offset of 5 mm from the edge of the antenna.

Following that, the reflection coefficient characteristics of the fabricated prototype are analysed and measured using the Agilent 8722ES Vector Network Analyzer, and the results are shown in Figure 18a, where the measured impedance bandwidth with S_11_ <−10 dB criterion is 15.3 GHz for a wideband FPRA ranging from 23.84 to 39.14 GHz, corresponding to a fractional impedance bandwidth of 48.58%. Despite minor differences, both simulation and measurement results are in good agreement, most likely due to assembly and fabrication errors. Thus, in the Mm-wave band spectrum, the proposed antenna performed outstandingly in terms of a wide impedance bandwidth.

Additionally, the proposed prototype’s peak gain, radiation patterns, and efficiency were evaluated in an anechoic chamber; the setup is shown in Figure 18b. By utilising the following equation, the gain of the antenna under test (AUT) is determined [36]:

(8) $$G_{AUT}=G_{horn}-P_{horn}+P_{AUT}$$

where $G_{horn}$ is the gain of the standard horn antenna. $P_{horn}$ and $P_{AUT}$ are the received power of the horn antenna (receiver mode) and the antenna under testing, respectively. A linearly polarised transmitting antenna measures the electric fields in two orientations, E-phi and E-theta, during the measurement of radiation patterns. The observed electric fields are then used to calculate the antenna’s left-hand electric field, right-hand electric field, and AR with both amplitude and phase [29]:

(9) $$E_{RHCP}=\frac{1}{\sqrt{2}}\left(E_{H}+jE_{V}\right)$$

(10) $$E_{LHCP}=\frac{1}{\sqrt{2}}\left(E_{H}-jE_{V}\right)$$

where $E_{H}=H_{a}cos\left(H_{p}\right)+V_{a}sin\left(V_{p}\right)$ and $E_{V}=H_{a}sin\left(H_{p}\right)-V_{a}cos\left(V_{p}\right)$

(11) $$AR=10\log(\frac{\left|E_{RHCP}\right|+\left|E_{LHCP}\right|}{\left|E_{RHCP}\right|-\left|E_{LHCP}\right|})$$

($H_{a}$, $V_{a}$) are the horizontal and vertical amplitude, and ($H_{p}$, $V_{p}$) are the phase components measured at each θ in the far-field.

**Figure 19:**
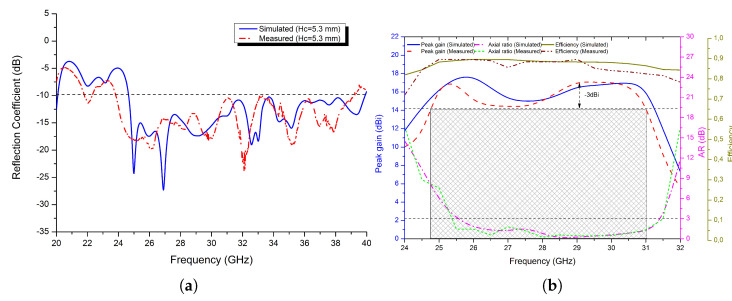
The simulated and measured (**a**) reflection coefficient of the proposed antenna and (**b**) peak gain and axial ratio at *h_c_* = 5.3 mm.

The results are depicted in Figure 19b, confirming that by using a PRS-based superstrate with a positively gradient phase, the peak gain over a wideband of a frequency spectrum is improved drastically while keeping the CP operation intact. From Figure 19b, the measured maximum peak gain of the proposed FPRA is 17,12 dBi at 29 GHz, with a −3 dB gain bandwidth of 22.42% (6,25 GHz) ranging from 24.75 to 31 GHz and an axial ratio of 21.75% (6,2 GHz) spanning over the spectrum of frequencies from 25.4 to 31.6 GHz. Moreover, the achieved radiation efficiency was 90%, which is in good agreement with the simulation results.

**Figure 20:**
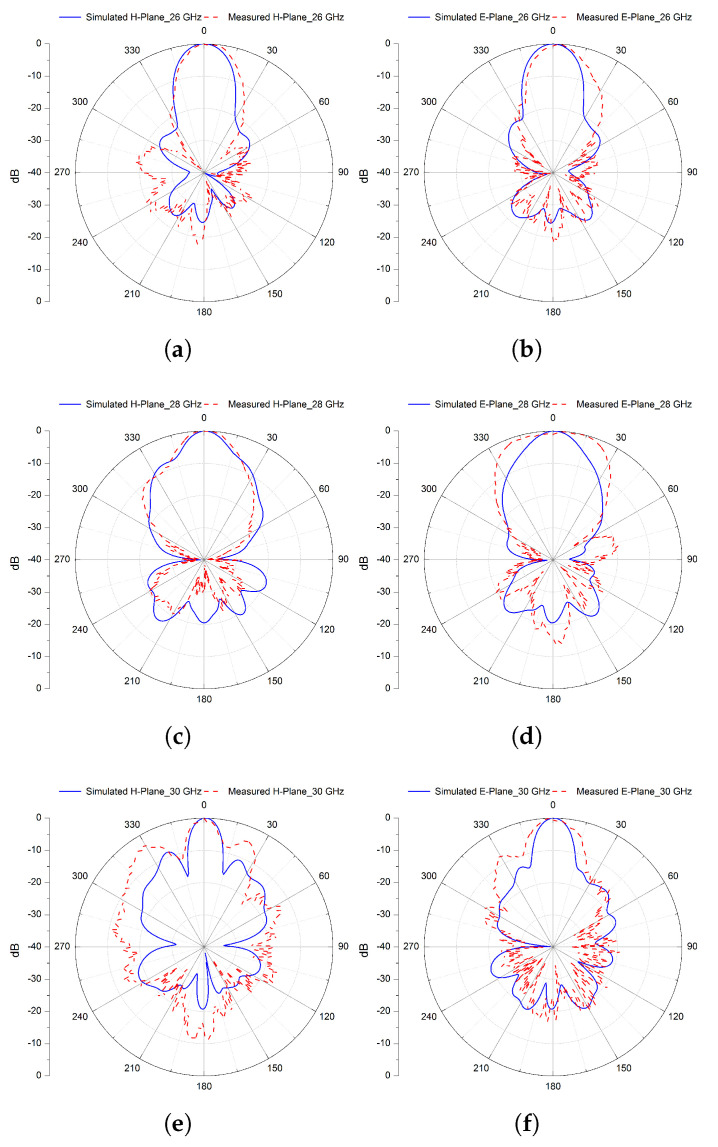
Normalised simulated and measured radiation patterns in the H− and E−planes at 26 (**a**,**b**), 28 (**c**,**d**), and 30 GHz (**e**,**f**) of the final 2 × 2 Sub-array CP antenna.

For further analysis, the normalised simulated and measured radiation patterns of the proposed FP resonator antenna are illustrated in Figure 20 at three different frequencies 26, 28, and 30 GHz, in both H− and E−planes. The obtained radiation patterns (simulated and measured) of the antenna are directional in the broadside of both the E− and H−planes, with lower side-lobe and cross-polarisation levels and a narrower beam-width.

Both simulation and measurement results are in good agreement, with a slight difference due to fabrication errors and assembly mismatching. With these features, the proposed design could be a potential candidate, allowing a high and consistent gain over a larger operating bandwidth and low profile for applications in the Mm-wave spectrum with circular polarisation capabilities.

Finally, the proposed FPRA design is compared to others in the literature, taking into account the bandwidth including the −3 dB gain bandwidth and the axial ratio one and the maximum achieved peak gain. The comparison is summarised in Table 4. From this comparison, it can be concluded that the proposed design outperforms the other ones in terms of high gain and wider operation bandwidth.

## 6. Conclusions

In this research, a topology optimised partially reflective surface (PRS) was created and simulated utilising the genetic algorithm implemented in Matlab, with a VBA-based interface established using CST Microwave studio. Because of the design symmetry, this proposed PRS design has a positive reflection coefficient phase, which results in a larger impedance, −3 dB gain bandwidth, and high gain at the Mm-wave band spectrum, ensuring polarisation independence for CP operation.

The proposed prototype was fabricated and tested. The simulation findings were corroborated by measurements, which revealed a wide−10 dB impedance bandwidth of 15.3 GHz extending from 23.84 to 39.14 GHz, corresponding to a fractional impedance bandwidth of 48.58%. Furthermore, the measured −3 dB gain bandwidth is 6.25 GHz (22.42%), ranging from 24.75 to 31 GHz, with a reported peak gain of 17.12 dBi, while the axial ratio was 21.75% (6.2 GHz). In addition, the achieved radiation efficiency was as high as 90% over the band of interest, and by using the proposed PRS layer, the surface current distribution was saturated and focused in comparison to the design without PRS.

Aside from the aforementioned enhancements, the proposed PRS layer altered the radiation pattern to become more directional, with a narrower beam-width and lower side-lobe levels. The simulation and measurement findings agree well, with a minor difference due to fabrication and assembly issues. The presented 2 × 2 sub-array PRS-based FP resonator antenna is a potential candidate for high-gain and wideband applications in the Mm-wave spectrum with circular polarisation capabilities. 

## Figures and Tables

**Table 1:** The slot-coupled patch antenna design parameters.

**Parameter**	***W_sub*2*_***	***w*_1_**	***w*_2_**	***l*_1_**	***l*_2_**
Value (mm)	10	2.4	0.9	10	3.3
**Parameter**	***l_*3*_***	***w_s_***	***l_s_***	***R_WP_***	***R_LP_***
Value (mm)	1.1	1.4	3.1	3.5	2.5

**Table 2:** Summarised results of the proposed design at different cavity spacings.

Cavity Spacing *h_c_* (mm)	|S11|<−10 dB BW (GHz)	3− dB Gain Bandwidth (GHz)	Cavity Spacing *h_c_* (mm)	|S11|<−10 dB BW (GHz)	−3 dB Gain Bandwidth (GHz)
5.3	27.6–32.8 (17.22%)	26.70–32.52 (19.65%)	5.7	25.81–32.05 (21.57%)	25.15–32 (24%)
5.4	27.15–32.54 (18.06%)	26.50–32.29 (19.73%)	5.8	25.45–31.95 (22.66%)	24.81–31.80 (24.69%)
5.5	26.66–32.33 (19.22%)	26.05–32.14 (20.95%)	5.9	25.10–31.90 (23.86%)	24.50-31.63 (25.42%)
5.6	26.2–32.17 (20.45%)	25.57–32.09 (22.60%)	6	24.77–31.5 (23.92%)	24.33–31.33 (25.15%)

**Table 3:** Parameter values of the parallel feeding network at 28 GHz.

***Z*_0_ (Ohm)**	58.9	63.3	78.1	66.2	72.8	50
**Width (mm)**	0.43	0.39	0.26	0.36	0.30	0.55
**Length (mm)**	1.73	1.74	3.5	3.48	1.75	Variable

**Table 4:** The proposed 2 × 2 sub-array design compared to other related works.

Ref. No	Antenna Type	S_11_ <−10 dB (%)	−3 dB Gain BW (%)	AR BW (%)	Max Gain (dBi)	Efficiency (%)
**[28]**	4 × 4 SIW	21.7	20.83	8.9	17.9	N/A
**[31]**	2 × 2 Parallel patch	38.1	N/A	24.15	12.89	85
**[38]**	4 × 4 SIW slot	4.6	6.56	10.7	16	96
**[39]**	2 × 2 DRA	33.8	10.16	5	9.5	N/A
**This work**	2 × 2 Parallel Elliptical patch	48.58	22.42	21.75	17.12	90

## Data Availability

Not applicable.
